# Author Correction: Ex vivo validation of magnetically actuated intravascular untethered robots in a clinical setting

**DOI:** 10.1038/s44172-025-00353-1

**Published:** 2025-02-03

**Authors:** Leendert-Jan W. Ligtenberg, Nicole C. A. Rabou, Constantinos Goulas, Wytze C. Duinmeijer, Frank R. Halfwerk, Jutta Arens, Roger Lomme, Veronika Magdanz, Anke Klingner, Emily A. M. Klein Rot, Colin H. E. Nijland, Dorothee Wasserberg, H. Remco Liefers, Pascal Jonkheijm, Arturo Susarrey-Arce, Michiel Warlé, Islam S. M. Khalil

**Affiliations:** 1 Department of Biomechanical Engineering, University of Twente, 7500 AE Enschede, The Netherlands; 2 Department of Design Production and Management, University of Twente, 7500 AE Enschede, The Netherlands; 3 Technical Medical Centre, University of Twente, 7500 AE Enschede, The Netherlands; 4 Radboud University Medical Center, 6525 GA Nijmegen, The Netherlands; 5 Department of System Design Engineering, University of Waterloo, ON N2L 3G1 Waterloo, Canada; 6 Department of Physics, German University in Cairo, New Cairo, 11835 Egypt; 7 LipoCoat B.V., 7521 AG Enschede, The Netherlands; 8 Laboratory of Biointerface Chemistry, TechMed Centre, University of Twente, 7500 AE Enschede, The Netherlands; 9 Mesoscale Chemical Systems, MESA+ Institute, University of Twente, 7500 AE Enschede, The Netherlands; 10 Department of Surgery, Division of Vascular and Transplant Surgery, Radboud University Medical Center Nijmegen, Nijmegen, The Netherlands

**Keywords:** Biomedical engineering, Mechanical engineering

Correction to: *Communications Engineering* 10.1038/s44172-024-00215-2, published online 16 May 2024

In the version of the article initially published, Michiel Warlé was incorrectly associated with the Department of Physics, German University in Cairo, New Cairo, 11835, Egypt. The correct affiliation is: Department of Surgery, Division of Vascular and Transplant Surgery, Radboud University Medical Center Nijmegen, Nijmegen, Netherlands. This has now been corrected in both the PDF and HTML versions of the article.

