# Supplementary material for: Ex vivo validation of magnetically actuated intravascular untethered robots in a clinical setting
Source: Commun Eng. 2024 May 16;3:68. doi: 10.1038/s44172-024-00215-2 (PMC11099159; doi:10.1038/s44172-024-00215-2)
Supplement: Supplementary file 2 — Supplementary materials [file 44172_2024_215_MOESM2_ESM.pdf]

Supplementary materials for:

Ex vivo Validation of Magnetically Actuated Intravascular Untethered Robots in a Clinical Setting

L.-J. W. Ligtenberg, N. Rabou, C. Goulas, W. C. Duinmeijer, F. R. Halfwerk, J Arens, R.Lomme, V. Magdanz, A. Klingner, E. A. M. Klein Rot, C. H. E. Nijland, D. Wasserberg, H. R. Liefers, P. Jonkheijm, A. Susarrey-Arce, M. Warlé and I. S. M.Khalil

This file includes:

Fig. S1 Flow response of a UMR with two fixed permanent magnets.

Fig. S2. Validation of coating presence on UMR material.

Fig. S3. UMR velocities in different actuation conditions and branch sizes in *ex vivo* settings.

Table S1. Arteries dimensions of the *ex vivo* models.

Table S2. Design parameters of the UMRs.

Legends for movies S1 to S4

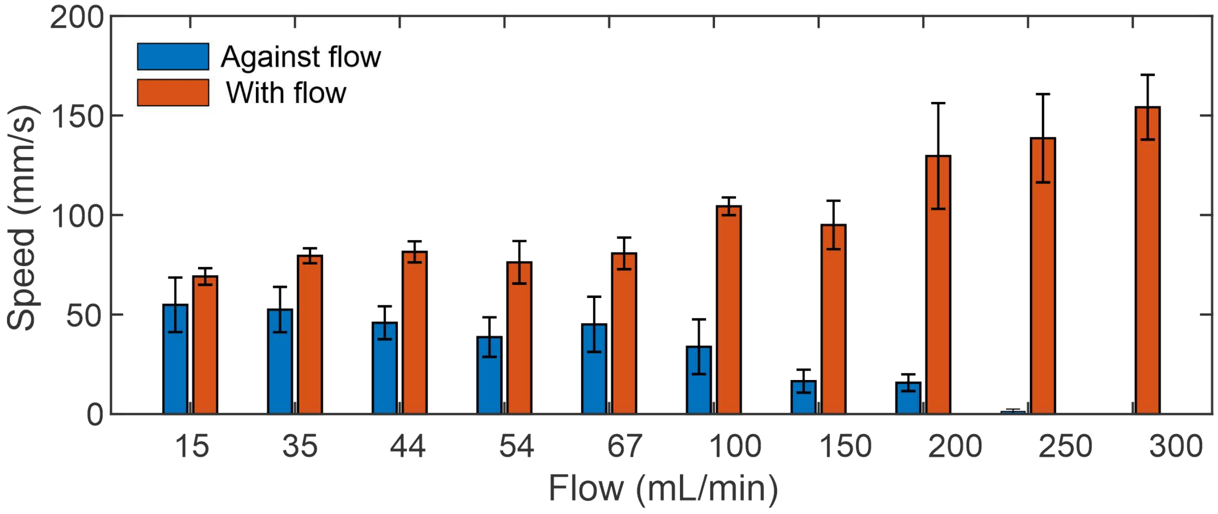

Fig. S1. A UMR equipped with two small permanent magnets can successfully swim against a flow rate of 250 mL/min. This UMR comprises a screw-shaped body and two cubic permanent magnets, each side measuring 1 mm.

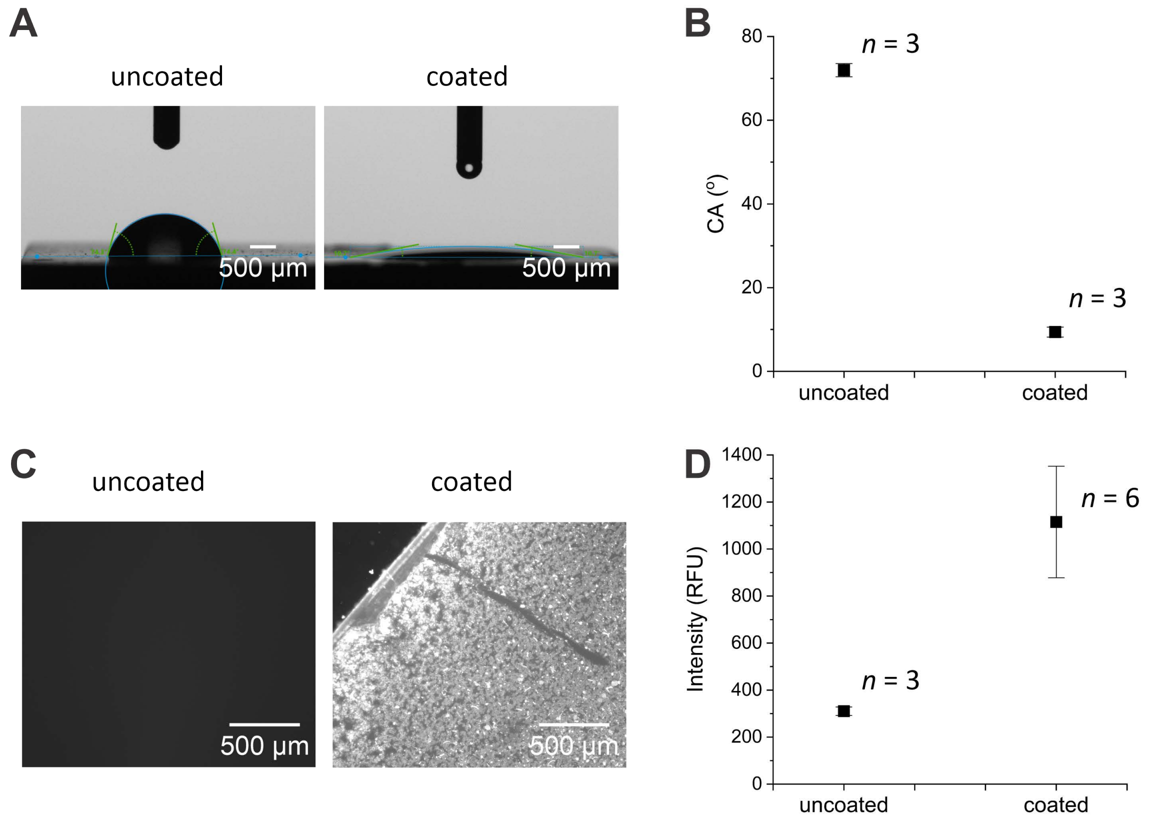

Fig. S2. Validation of coating presence on UMR material is depicted as follows: (A) Images display sessile drops of deionized water from contact angle measurements on uncoated and coated samples. (B) Average contact angle values are presented. (C) Micrographs show UMR material without and with fluorescently-labeled coating, with images representing a 2173 µm x 1818 µm (3.95 mm²) area. (D) Averaged fluorescence intensity of UMR material without and with fluorescently-labeled coating is illustrated.

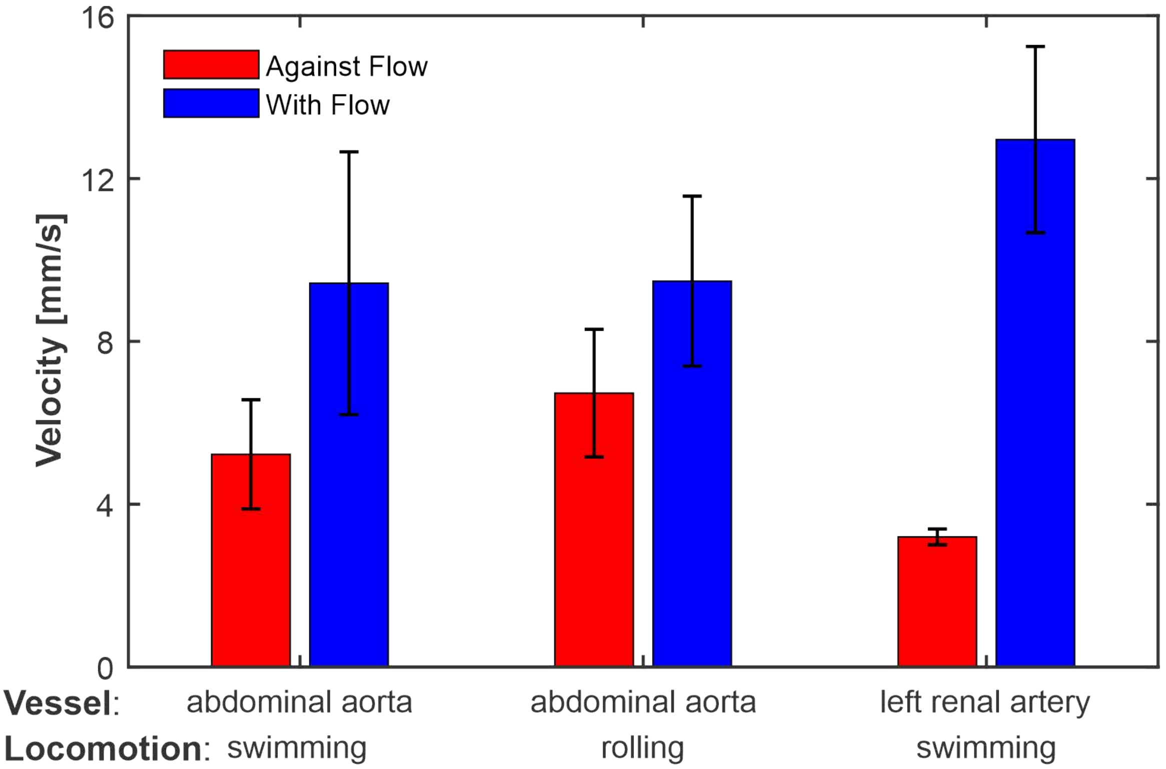

Fig. S3. The velocities of the UMR against and with flow (15 mL/min) during swimming and rolling in various arteries, such as the abdominal aorta and left renal artery, are evaluated under *ex vivo* conditions.

Table S1. Artery dimensions of the *ex vivo* models

Blood vessel	Inner diameter [mm]
Abdominal aorta	9.8-14.7
Left renal artery	4.2-6
Right renal artery	4.6-6

Table S2. Design parameters of the UMRs

UMR	12-mm-long	9-mm-long
Length [mm]	12.66	9.495
Diameter [mm]	5	3.75
Inner cylinder diameter [mm]	1.2-2.5	0.9-1.875
Fin thickness [mm]	0.5	0.375
Fin pitch [mm]	3.6-12	2.9-9
