# Supplementary material for: Ex vivo validation of magnetically actuated intravascular untethered robots in a clinical setting
Source: Commun Eng. 2024 May 16;3:68. doi: 10.1038/s44172-024-00215-2 (PMC11099159; doi:10.1038/s44172-024-00215-2)
Supplement: Supplementary file 3 — Description of Additional Supplementary Files [file 44172_2024_215_MOESM3_ESM.pdf]

Description of Additional Supplementary Files

File name: Supplementary Movie S1

Description: Magnetic actuation of a UMR in the Abdominal aorta and left renal artery of an ex vivo perfusion model

File name: Supplementary Movie S2

Description: UMR swimming trajectories evaluated across a blood flow range of 15 to 67 ml/min in an ex vivo abdominal aorta

File name: Supplementary Movie S3

Description: UMR locomotion mechanism under the influence of a rotating magnetic field and out-of-plane magnetic torque

File name: Supplementary Movie S4

Description: Ex vivo navigation of UMRs through the abdominal aorta, including a turning maneuver into the left renal artery
